# Direct Inhibition of β-catenin: A new strategy for colorectal cancer

**DOI:** 10.18632/oncoscience.477

**Published:** 2019-01-31

**Authors:** Shanthi Ganesh, Bob D. Brown, Marc Abrams

**Affiliations:** Dicerna Pharmaceuticals, Inc, Cambridge, MA 02140, USA

**Keywords:** Wnt/β-catenin pathway, Colorectal Cancer, RNAi therapeutics, Resistance, Combinations

Colorectal cancer (CRC) is the third most commonly diagnosed cancer among both men and women in the United States and remains an area of significant clinical need. Inactivation of the *APC* tumor suppressor gene is considered an initiating event and the key oncogenic driver in most CRCs; disruption of APC protein function drives activation of the Wnt/β-catenin signaling pathway. CRCs also harbor a high incidence of other mutations that often arise as a secondary event after Wnt/β-catenin dysregulation (multistep somatic evolution model) such as in *KRAS*, *BRAF*, *TP53*, *P13K* and/or *SMAD4* that cooperate with *APC* loss to further drive tumor progression [1]. Treatment regimens for advanced unresectable CRC involve combination chemotherapies that are not curative. Current-generation targeted therapies including EGFR, VEGF and VEGFR inhibitors, have achieved mixed results. EGFR inhibitors for example, do not benefit patients with *KRAS/BRAF* mutations and yield a relatively low response rate. MEK inhibitors are also being explored as a treatment for patients with *KRAS/BRAF* mutant tumors who are not candidates for EGFR-directed therapies, however, these compounds have yielded limited efficacy to date. A triple-combination regimen of BRAF, MEK, and EGFR inhibitors showed improved progression free survival (PFS) compared to the individual agents, but even this potent cocktail yielded a PFS of only 4.2 months in refractory CRC [2]. Rapid emergence of drug resistance and dose-limiting toxicities have delayed the pace of clinical progress.

Given the status of Wnt/β-catenin signaling as the primary driver of most CRCs and its role as a resistance mechanism in *KRAS/BRAF* mutant CRCs [3], adding Wnt/β-catenin pathway inhibition to current standard-of-care may be a broadly applicable strategy to increase response rates in this disease. Unfortunately, no therapies specifically targeting this pathway have achieved regulatory approval. Targeting β-catenin protein directly, although arguably the most logical approach, is extremely challenging using conventional drug modalities such as small molecules. Indirect approaches to reduce Wnt/β-catenin signaling have included targeting upstream Wnt receptors, Wnt ligand secretion, or protein complexes containing β-catenin, but have also had limited success due to signaling pathway redundancy, heterogeneity, and clinical toxicities. For example, targeting Wnt ligand/receptor biology, including the well-studied inhibitors of the PORCN O-acyltransferase, may be effective only against relatively low-occurrence tumors that with specific mutations upstream of *APC* (*RSPO*, *LKB1*, *RNF43*). Tankyrase (TNKS) inhibitors, which are believed to act by countering the effects of APC loss on β-catenin stability, may be more broadly applicable but affect a number of unrelated cellular processes. The broad biodistribution of TKNS and similar inhibitors also increases the potential for mechanism-based toxicities associated with Wnt/β-catenin inhibition in normal tissues including intestine and bone marrow [4]. Several groups have developed promising strategies to block the interaction of β-catenin with transcriptional coactivators, but it is possible similar safety concerns will emerge. Overall, targeting β-catenin directly in the tumor, while limiting the exposure in normal tissues, appears to be the most promising and yet most challenging approach to treating CRC.

Nucleic acid-based therapeutics such as antisense and RNA interference (RNAi) have been studied in oncology trials for almost 30 and 15 years, respectively, with limited success. However, recent regulatory approvals in diverse non-oncology indications may rekindle interest in these approaches in oncology research. RNAi, which bypasses the challenge of drugging the β-catenin protein by targeting β-catenin mRNA, has demonstrated favorable results in multiple CRC preclinical models. Furthermore, RNAi drug products generally lack broad intracellular delivery to non-hepatic tissues, potentially limiting mechanism-based off-target effects. While several early clinical trials using first-generation RNAi drug products for oncology applications have faltered, recent regulatory approvals and striking advances in both delivery technology and oligonucleotide chemistry have increased the potential for future success. In one example of this approach, our laboratory has developed DCR-BCAT, a chemically-optimized Dicer-substrate siRNA (DsiRNA) formulated in a tumor-selective lipid nanoparticle (LNP) [5]. The lipid components of DCR-BCAT have been screened and selected systematically to enhance accumulation and internalization into preclinical tumors, as demonstrated in diverse preclinical models, while limiting functional delivery and sparing normal β-catenin function in most normal tissues. The notable exception is the liver, where β-catenin reduction is observed, but the level of reduction is not sufficient to yield a toxic phenotype [5]. The potent and selective RNAi-mediated reduction in β-catenin levels by DCR-BCAT in preclinical models resulted in anti-tumor efficacy in tumors with both *APC* and other Wnt/β-catenin pathway mutations. DCR-BCAT in combination with MEK inhibition (MEKi) also displayed synergistic efficacy and overcame MEKi-mediated drug resistance [3].

Finally, it is worth noting that recent years have uncovered an important role of β-catenin in tumor immunology. Wnt/β-catenin signaling functions to exclude cytotoxic T-cells from accumulating in tumors, therefore causing resistance to immune checkpoint inhibitors [6]. Wnt signatures are inversely correlated with tumor infiltrating lymphocytes (TILs) in CRCs [7]. Non-hypermutated microsatellite stable (MSS) CRCs, which comprise ~85% of all CRCs, are associated with low response rates to immunotherapy [7] and Wnt dysregulation has been strongly implicated. In preclinical models, DCR-BCAT significantly improved T-cell infiltration and responses to PD-1 and CTLA-4 checkpoint inhibition in both Wnt-active and non-Wnt-active tumors [8]. These observations suggest another mechanism by which tumor-selective β-catenin inhibition may improve outcomes.
